# 

**Published:** 1919-03

**Authors:** 


### THANK YOU.

We hereby acknowledge receipt of subscriptions from the following persons.

| | |
|---|---|
| Dr. H. H. Beverly, Smiley, Texas | Paid to July 1919 |
| Dr. A. Beckman, Bartlett, Texas | Paid to Oct. 1919 |
| Dr. J. W. Bond, Donie, Texas | Paid to Dec. 1919 |
| Dr. W. D. Cross, Corsicana, Texas | Paid to June 1920 |
| Dr. R. K. Ferguson, Miles, Texas | Paid to July 1920 |
| Dr. J. E. Cannon, Celeste, Texas | Paid to June 1920 |
| Dr. M. E. Daniel, Honey Grove, Texas | Paid to July 1919 |
| Dr. Z. L. Daniel, Gary, Texas | Paid to Oct. 1920 |
| Dr. J. L. Ellison, Beaumont, Texas | Paid to May 1920 |
| Dr. J. E. Fuller, Paris, Texas | Paid to Sept. 1920 |
| Dr. J. J. Gill, Dodd City, Texas | Paid to June 1920 |
| Dr. C. E. Hall, Lindale, Texas | Paid to May 1919 |
| Dr. I. M.Howard, Burkett, Texas | Paid to May 1919 |
| Dr. R. F. Harnesburger, Beckville, Texas | Paid to May 1920 |
| Dr. A. W. Irwin, Floresville, Texas | Paid to June 1919 |
| Dr. E. Johnson, Montague, Texas | Paid to July 1919 |
| Dr. T. T. Jackson, San Antonio | Paid to March 1919 |
| Dr. T. J. Knight, Ravenna, Texas | Paid to Oct. 1919 |
| Dr. G. T. King, Elgin, Texas | Paid to July 1919 |
| Dr. J. J. Lockhart, Wells, Texas | Paid to July 1920 |
| Dr. J. D. Leonard, Bronte, Texas | Paid to Dec. 1920 |
| Dr. W. W. Latham, Crockett, Texas | Paid to May 1920 |
| Dr. J. D. Mickie, Childress, Texas | Paid to Feb. 1919 |
| Dr. J. A. Daniel, Carthage, Texas | Paid to Jan. 1920 |
| Dr. J. R. Myrick, Novice, Texas | Paid to June 1920 |
| Dr. W. A. Morrow, Tulia, Texas | Paid to May 1920 |
| Dr. R. Menger, San Antonio, Texas | Paid to Sept. 1919 |
| Dr. O. H. Neighbors, Seguin, Texas | Paid to July 1920 |
| Dr. C. M. Rosser, Dallas, Texas | Paid to Nov. 1920 |
| Dr. T. R. Pettway, Austin, Texas | Paid to Sept. 1919 |
| Dr. J. G. Saddler, Henderson, Texas | Paid to Dec. 1920 |
| Insane Asylum, Austin, Texas | Paid to Sept. 1919 |
| Dr. A. Ross, Lockhart, Texas | Paid to Dec. 1919 |
| Dr. E. M. Thomas, Georgetown, Texas | Paid to March 1920 |
| Dr. R. E. Wright, Winnsboro, Texas | Paid to May 1920 |
| Dr. J. P. Wier, Covington, Texas | Paid to Oct. 1919 |
| Dr. C. W. Welch, Caddo Mills, Texas | Paid to July 1922 |
| Dr. W. C. Windham, Shelbyville, Texas | Paid to June 1919 |
| Dr. V. L. Van Dyke, Detroit, Texas | Paid to Oct. 1920 |
| Dr. B. A. Ziegler, Shamrock, Texas | Paid to Dec. 1920 |
| Dr. J. M. Parker, Teneha, Texas | Paid to Oct. 1920 |
| Dr. J. D. Griffith, Kansas City, Mo | Paid to Dec. 1919 |
| Dr. R. W. Barton, Marion, Arkansas | Paid to Oct. 1920 |
| Dr. H. T. Jones, Almyra, Ark | Paid to Nov, 1920 |
| Dr. Guido Ranniger, Oscuno, N. M | Paid to June 1920 |
| Dr. J. G. Moir, Deming, N. M | Paid to June 1919 |
| Dr. R. L. Parrott, Zwolle, La | Paid to Oct. 1920 |
| Dr. J. E. Crawford, Ludington, La | Paid to Oct. 1919 |

### Old Jokes That We Have Met.

**Recognized Her Kind.**

A director of one of the great transcontinental railroads was showing his 3-year-old daughter the pictures in a work on natural history. Pointing to a picture of a zebra, he asked the baby to tell him what it represented. Baby answered, "Coty."

Pointing to the picture of a tiger in the same way, she answered, "Kitty." Then a lion, and she answered, "Doggy." Elated with her seeming quick perception, he then turned to the picture of a chimpanzee, and said:

"Baby, what is this?"

"Papa."

---

**The Expression.**

By contorting the face epidermis,
Or skin, or whatever the term is,
We express or display
Pride, joy or dismay,
The expression's wherever the squirm is.

—*The Limeratomy.*

---

**Caution Man.**

"You must stand in front of an open window every morning and take deep breaths."

"I can't do that, Doctor."

"Why not?"

"I have only one window in my room. That faces the apartment of an old maid. I don't want her to get the idea that I'm trying to start something."—*Birmingham Age-Herald.*

---

**Mistakes Will Happen.**

A woman doctor of Philadelphia was calling on a young sister, recently married, who was in distress. In response to the doctor's inquiry, the newly wed said:

"I cooked a meal for the first time yesterday, and I made an awful mess of it."

"Never mind, dearie," said the doctor, cheerfully; "it's nothing to worry about. I lost my first patient."—Harper's.

---

Natural Affinity:—There seems to be a strange affinity between a colored man and a chicken, a Northern man once remarked to Senator Morris Sheppard of Texas.

"There's nothing strange about that," replied Mr. Sheppard, "one is descended from Ham and the other from Eggs."

---

"Doctor," said he, "I'm a victim of insomnia. I can't sleep if there's the least noise, such as a cat on the back fence, for instance."

"This powder will be effective," replied the physician, after compounding a prescription.

"When do I take it, doctor?"

"You don't take it. You give it to the cat, in a little milk."

---

**His Handiwork.**

"What a distinguished looking man your father is! His white hair gives him such an aristocratic look."

"Yes," said the distinguished looking man's son, "he can thank me for that."

---

**A Dependable Guide.**

Friend—What is the first thing you do when a man presents himself to you for consultation?"

Doctor—I ask him if he has a car.

Friend—What do you learn from that?

Doctor—If he has one, I know he is wealthy—and if he hasn't I know he is healthy.—Buffalo Courier.

---

**Mother Goose Revised.**

Hear! Hear!
  The germs are near;
Pneumonia taints the breeze.
  It enters and grows
  In the mouth and the nose,
And spreads through a cough and a sneeze.

---

Nearly everybody nowadays appears to be in favor of Government ownership of something if it belongs to somebody else. —New York World.

---

"Dear mother," he wrote, "Ernest swallowed a dime yesterday and we have been much worried ever since as to whether or not he is going to be ill." Then he addressed the letter to Boston. A week later the old lady replied:

"My dear son," she began, "I have been unable to rest since hearing from you last. Please let me know as soon as you can if Ernest got over his financial difficulties alright.''

---

**The Optimist.**

Doctor (to patient with broken leg)—How are you this morning?

Patient.—Oh, can't kick.

---

**His Name.**

''What is your name?'' a Kentuckian asked of a small negro boy.

''Well, boss,'' answered the chap, ''everywhere I goes dey gibs me a new name but mah maiden name was Moses.''

---

**Skill.**

Gingham—Do you consider Dr. Seton a skillful physician?

Butcher.—None better in town. Pays his bills regular.

---

**Diagnosis.**

Mrs. Casey.—The docthor says ye hov appendikitis, Tim!

Mr. Casey—Och, Norah, Norah! Whoy wor ye so foolish as to show him yure bank book?

---

The lawyer was trying very hard for his client and was setting the points out in a logical manner. There was one thing he was note quite clear about and he accordingly said:

''Now, sir, you state my client knocked you down and then disappeared in the darkness. What time of night was this?''

''I can't say exactly,'' the complainant answered, dryly. ''Your client had my watch.''—New York Globe.

---

Most people's reply to the suggestion to eat horse flesh is an indignant ''Neigh!''—London Opinion.

A Lucky Discovery—Two women of the parvenu class were discussing the future of their respective sons, when one of them them said:

''Do you know, I believe that a boy's development depends largely upon his environment?''

''I know it,'' replied the other, as she carelessly toyed with her jewel box. ''There was my cousin William's boy he never knew what it was to have a well day till the doctor found out that the trouble was with his environment and cut it out.''—Harper's.

FRIEND—"Doctor, how do you manage to stand the high cost of living?"

SURGEON—"By cutting out something."—Brooklyn Citizen.

